# Directional hearing: from biophysical binaural cues to directional hearing outdoors

**DOI:** 10.1007/s00359-014-0939-6

**Published:** 2014-09-18

**Authors:** Heiner Römer

**Affiliations:** Institute of Zoology, Karl-Franzens University Graz, Universitätsplatz 2, 8010 Graz, Austria

**Keywords:** Directional hearing, Pressure difference receiver, Binaural cues, Interaural time difference, Interaural intensity difference

## Abstract

When insects communicate by sound, or use acoustic cues to escape predators or detect prey or hosts they have to localize the sound in most cases, to perform adaptive behavioral responses. In the case of particle velocity receivers such as the antennae of mosquitoes, directionality is no problem because such receivers are inherently directional. Insects equipped with bilateral pairs of tympanate ears could principally make use of binaural cues for sound localization, like all other animals with two ears. However, their small size is a major problem to create sufficiently large binaural cues, with respect to both interaural time differences (ITDs, because interaural distances are so small), but also with respect to interaural intensity differences (IIDs), since the ratio of body size to the wavelength of sound is rather unfavorable for diffractive effects. In my review, I will only shortly cover these biophysical aspects of directional hearing. Instead, I will focus on aspects of directional hearing which received relatively little attention previously, the evolution of a pressure difference receiver, 3D-hearing, directional hearing outdoors, and directional hearing for auditory scene analysis.

## Introduction

In insect taxa which use acoustic signals for intraspecific communication, the recognition of the acoustic signals is crucial for their fitness, since it enables species identification and prevents hybridization (Gerhardt and Huber 2002; Greenfield 2002; Bradbury and Vehrencamp 2011). However, ears also evolved in insects without acoustic signals, indicating that other functions of hearing may also include the detection of cues from predators, or the detection of hosts in the case of parasitoids. The anatomical and physiological diversity of insect ears we know so far is impressive, with perhaps 19 independent evolutionary origins, as is the diversity of body parts on which they evolved, including legs, wings, mouth parts or the abdomen (Hoy and Robert 1996; Yager 1999; Yack 2004).

The identification of the acoustic signal as species-specific, or the discrimination between different signal variants, is only one part of the task: the signal should also be correctly localized. The negative fitness consequences for a prey escaping into the wrong direction in the face of a predator are obvious, similar to wrong or distorted directionality in the process of finding and approaching a mate. Therefore, we should expect that the evolution of these ears and the neuronal network processing the directional cues enabled insects to localize a sound source reasonably well. In the case of particle velocity receivers such as the antennae of mosquitoes, or filiform hairs on the cerci directionality is no problem because such receivers are inherently directional. They respond to the particle velocity (and thus a vectorial) component of near-field sound.

The problem with the task of directional hearing is obvious, however, with tympanate hearing; it is basically a biophysical one associated with the small size of most insects. Insects equipped with bilateral pairs of tympanate ears could principally make use of binaural cues for sound localization, like all other animals with two ears. However, their small size and hence interaural distance result in only minute interaural time differences (ITDs). At the same time acoustic theory predicts that significant diffraction for the establishment of reasonable interaural intensity differences (IIDs) occurs only when the ratio of body size to the wavelength of sound exceeds a value of 0.1 (Morse and Ingard 1968; Robert 2005). Again, the small body size of insects in relation to the relatively large wavelength of the sound signals used for communication thus often limits or even prevents the establishment of reasonably large IIDs through diffraction.

In my review, I will only shortly cover the biophysical aspects of directional hearing; more complete descriptions of the biomechanics of sound propagation and the generation of cues for directional hearing in insects can be found in excellent earlier reviews (Michelsen 1992, 1998; Robert and Hoy 1998; Robert 2005 for all kind of receivers; Michelsen and Larsen 2008 for pressure difference receivers). Instead, after shortly reviewing the different kind of receivers in insects, I will focus on aspects of directional hearing which received relatively little attention in previous reviews, namely the evolution of a pressure difference receiver, 3D-hearing, directional hearing outdoors, and directional hearing for auditory scene analysis.

## Pressure and pressure difference receivers, and mechanically coupled receivers

If sound can act on only one side of the tympanal membrane, ears operate as pure pressure receivers. When insects are large compared to the wavelength of the sound frequencies of relevant signals, the insect’s body can generate diffractive effects, resulting in a reduced pressure at the contralateral ear. IIDs as large as 40 dB have been reported for a large noctuid moth with ultrasonic frequencies used by echolocating bats (Payne et al. 1966). Compared to the minimum values of about 1 dB necessary for reliable directional responses (see below) such IIDs are rather large.

In pressure difference receivers, sound can act on both sides of the tympanal membrane, requiring two (or even more) acoustic inputs which conduct pressure waves also to the internal side of the tympanum. Thus, the force driving the tympanal membrane is the difference between the external and internal pressures. Since the internal pressure component travels either through some body tissue, air sacs or in tracheal tubes, various degrees of attenuation or amplification and phase shift can occur relative to the external component. Theoretically, evolutionary modifications of anatomical structures (see below) resulting in the proper amplitude and phase shifts between internal and external pressure components could create highly directional ears despite unfavorable ratios of body size to the wavelength of sound. Since the internal sound conduction can depend on frequency, insect ears can act as pressure difference receivers at low frequencies and as a pressure receiver at higher frequencies. The locust (grasshopper) ear is such a case (Michelsen and Rohrseitz 1995; Schul et al. 1999). Together with the other well-studied pressure difference receiver, the cricket ear (see below), the grasshopper example highlights the importance of proper phase relationships between the external and internal sound component(s) for producing significant directionality.

The ears of the tachinid fly *Ormia ochracea* are an example for mechanically coupled ears. They are so close together that both IIDs and ITDs appear to be much too small for a physical basis of directional hearing. For example, the interaural distance of 520 µm would create no more than 1.45 µs of ITD, and significant diffractive effects cannot play a role given the unfavorable ratio of body size to wavelength of sound (Robert et al. 1996). Despite these limitations, the flies show very accurate acoustic localization behavior in flight (see below) and while walking (Mason et al. 2001; Müller and Robert 2001). The mystery of the directionality of these ears has been solved by the finding that due to the mechanical coupling of the tympana by a flexible cuticular lever the two tympanal membranes move out of phase at frequencies relevant for the fly (Miles et al. 1995; Robert et al. 1996). The mechanical ITD of 50–60 µs thus created is much larger than the acoustical ITD of 1.45 µs. The larger interaural differences can then be processed by the nervous system (for a detailed review on how these mechanical cues are used for the reliable neural coding of sound direction see Robert and Göpfert 2002; Robert 2005).

A necessary word of caution should emphasize that the actual strategy of finding a host in nature may be more complex, as shown for another parasitoid fly *Emblemasoma auditrix* (Lakes-Harlan and Köhler 2003). In an open area, more than half of the animals flew directly to the loudspeaker (broadcasting a model song of their cicada host), but the presence of single landmarks reduced the percentage of direct flights to the target greatly. Flies used these landmarks to stop and re-orientate towards the loudspeaker. Future studies will show whether this reflects species differences or the influence of a more or less complex structured environment.

## Minimum binaural cues for sound localization

**Fig. 1:**
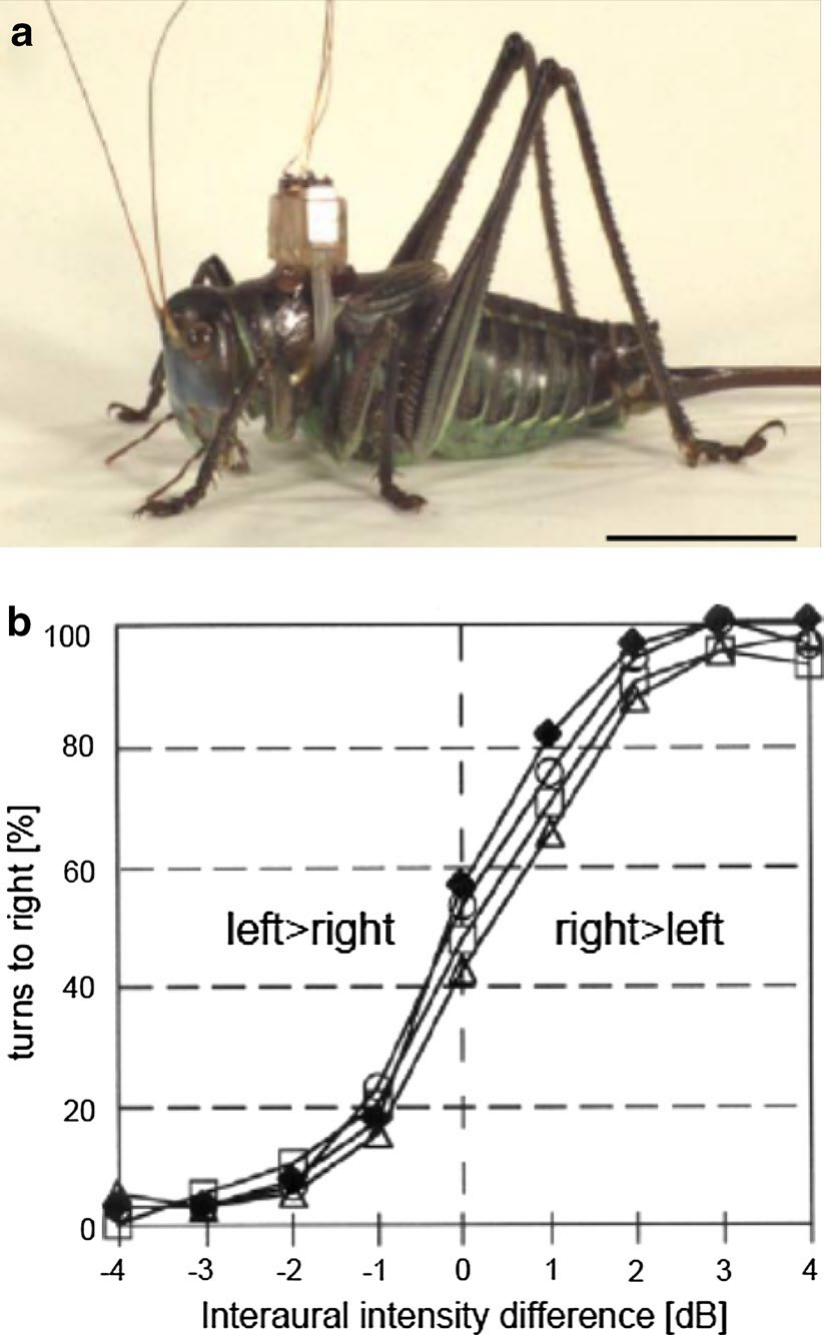
**a** Female *Gampsocleis gratiosa* with a dichotic ear stimulation device. A backpack carrying two miniature speakers were attached to the dorsal pronotum. Each speaker is connected via plastic tubes to the large spiracular opening of the acoustic trachea of the respective ipsilateral side, so that sound can be separately applied through the leg trachea to the inner side of each ear. *Scale bar* = 2 cm. **b** Results of behavioural dichotic stimulation experiments with four females, in which the interaural intensity difference of a stimulus was varied. Note that with an IID of 1 dB there is a significant turn to the more strongly stimulated side, and with 2–3 dB difference very few incorrect turns are made (from Rheinlaender et al. 2005)

Insects usually base their phonotactic approaches on a binaural comparison. Whatever the biophysical mechanism creating binaural cues for sound localization, what are the minimum IIDs and ITDs that result in reliable directional responses in insects? In dichotic experiments one can apply lateralized stimuli to either hearing organ without stimulating the opposite one in a wide range of intensities, so that the minimum interaural cues involved in localization can be tested. This is a difficult task in insects when body size and hence interaural disparities are minute and/or the two hearing organs are acoustically coupled. A tentative behavioral approach with a grasshopper indicated that the insects’ auditory system can make use of IIDs in the order of 1–2 dB (von Helversen and Rheinlaender 1988). Using a dichotic ear stimulation device for freely moving katydid females (cross-talk barrier of about 50 dB), females turned to the stronger stimulated side starting with a 1 dB difference between both ears (Rheinlaender et al. 2005; Fig. 1). The ability to resolve such small IIDs corroborates quantitative data on the accuracy of phonotactic approaches of the same insect, where females may experience only small stimulus angles of 6–10°, which create only small IIDs in the order of 1–2 dB, but still make significant correct turns. The directionality of phonotaxis in female *G. bimaculatus* walking on an open-loop trackball system indicated even smaller interaural differences for reliable lateralization (Schöneich and Hedwig 2010; see below).

But what about the use of ITDs as a cue for sound localization? The interaural disparity in insects can be extremely small, hence the available ITDs amount to only 3–5 µs (short-horned grasshoppers), or only 1.45 µs (parasitoid fly). These values of ITDs are so strongly constrained by body size and ear separation, that it had been accepted for a long time that such small ITDs cannot be used for neuronal processing of sound direction. Mörchen (1980) thus suggested that insects do not use the physical ITDs, but the physiological time differences in the binaural receptor fibre discharges instead. He examined in locust auditory receptor fibres the effect that the latency of sensory excitation is dependent on stimulus intensity (Imaizumi and Pollack 2001 for a similar finding in crickets). The high negative correlation between response strength and latency results in a direction-dependent latency shift in the afferent activity. Thus, the difference in the time of arrival of action potential activity on both sides of the nervous system may reach values of 5–6 ms for ipsi-versus contralateral sound, which exceeds the value of the physical ITD between the ears by almost 1,000 times. The author therefore suggested that response strength and response latency can be regarded as equivalent directional cues for sound direction. Similarly, in the study by Schöneich and Hedwig (2010) the interaural intensity differences were closely reflected in the response latencies of the auditory afferent activity. The overall bilateral latency difference at an angular deviation of 30° was 1.28 ms whereas the actual interaural difference in ITD is less than 15 µs.

The hypothesis of the dual mode of directional coding was examined using dichotic stimulation of the tympana in the locust (Rheinlaender and Mörchen 1979). They demonstrated a time-intensity trading phenomenon similar to the one reported for vertebrates (Erulkar 1972), although the time cue was in the order of milliseconds, rather than microseconds as indicated by the physical ITDs. Shifting the contralateral stimulus only 2–4 ms ahead of the ipsilateral one could drive the activity of an interneuron from maximal excitation into total inhibition. In the quasi-dichotic stimulus situation mentioned above, the behavioral resolution of *Chorthippus biguttulus* males for ITDs was also in the order of 1 ms: when both stimuli were presented at equal loudness, but one speaker was leading the other by only 0.5 ms this resulted in significant turns towards the leading side (von Helversen and Rheinlaender 1988). All together, these experiments confirm that the dual mode of directional coding provided by auditory receptor fibres is indeed used at the first site of synaptic processing. Although microseconds do not matter because the physical time delays between the ears are too small to be of relevance (but see results for the *Ormia* fly below), the onset of binaural arrival of receptor activity at these synapses (the physiological time delay) may be quite important for directional coding.

## IIDs and ITDs as represented at the neuronal level

Whatever the magnitude of these binaural differences: How are behaviorally relevant ITDs and IIDs encoded in the discharge differences of afferents and interneurons of the auditory pathway? The high behavioral precision discussed above for some acoustic insects is surprising when considering the low number of receptors in each ear and the high variability of receptor responses (Ronacher and Krahe 2000). Moreover, even fewer elements are available at the level of interneurons. In their attempt to determine the neuronal correlates of such small IIDs for grasshoppers, Ronacher and Krahe (2000) noted that an IID of 1.5 dB corresponds to a spike count difference of only 1 spike per response (for a stimulus of 100 ms) in a bilateral pair of receptors, but that the error probabilities for a decision based on these differences are larger than those observed in behavior. Their conclusion was that the insect has to integrate information from up to 13 receptors to arrive at the observed behavioral precision. The prediction has been tested indirectly by Stradner and Römer (2008) for a pair of first-order local interneurons (omega-neurons) in katydids, which integrate excitatory inputs from most of the auditory receptors in the ipsilateral ear (Römer et al. 1988), and inhibitory inputs from the contralateral side mediated by its mirror-image counterpart on the other side (Selverston et al. 1985; Molina and Stumpner 2005). Using an independent ear stimulation paradigm Stradner and Römer (2008) demonstrated that starting with an IID of 1 dB, the discharge differences in this pair of interneuron were large and significant, with the louder side being more strongly excited. Thus small, behaviorally relevant IIDs are available for these insects from simple spike count differences in pairs of auditory interneurons (for detailed reviews of neural processing of directional information see Hennig et al. (2004), and Hedwig and Pollack (2008).

The comparison of the two species with hyperacute directional hearing, the field cricket *G. bimaculatus* and the fly *Ormia ochracea* may illustrate the limits of processing binaural cues for directional hearing known so far. Both species have been tested under ideal acoustic conditions under open-loop conditions on a trackball (Mason et al. 2001; Schöneich and Hedwig 2010). Females of both species not only reliably discriminated the side of acoustic stimulation at angles of sound incidence starting 1–2° from the animal’s longitudinal body axis (indicative of correct lateralization), but also precisely walked towards the sound source. In the cricket study, the tympanic membrane oscillations of the ears revealed between 0 and 30° a linear increasing function of interaural amplitude differences with a slope of 0.4 dB/°. Such small IIDs were closely reflected in the bilateral latency difference of responses of auditory afferents, which was 1.28 ms compared with the physical ITD of less than 15 µs (Schöneich and Hedwig 2010).

The linear latency gradient of 42 µs/° in the frontal zone of the female cricket appears to be small, but the *Ormia* fly even exploits a gradient of only 3.5 µs/°. As mentioned above, in the fly’s ear the interaural distance of 520 µm would create no more than 1.45 µs physical ITD. However, these ears are mechanically coupled pressure receivers. The coupling of the tympana has the effect that the two tympanal membranes move out of phase at the CF of the cricket song, which is the relevant frequency for the parasitoid fly (Miles et al. 1995; Robert et al. 1996). The mechanical ITD of 50 µs is much larger than the acoustical ITD of 1.45 µs. However, with the small stimulus angles they can discriminate in the frontal zone, the flies have to deal with acoustical ITDs as small as 50 ns, and 2 µs for the mechanical ITD. To process such small time differences adequately, the primary afferents exhibit properties different from those of other acoustic insects (Oshinsky and Hoy 2002). They are not spontaneously active, and respond with one action potential to acoustic stimuli with very high temporal acuity. The variation in the timing of the single spike was remarkably low with 95 µs. And similar to the findings in locusts and crickets (see above), there is an inverse relationship between the latency of the response and stimulus amplitude. In this way, physiological time differences in spike timing are generated between ipsi-and contralateral afferents with a magnitude of about 600 µs, again much larger than the physical ITDs (Oshinsky and Hoy 2002; review in Robert 2005).

## The evolution of the pressure difference receiver in crickets

The anatomical basis for a functional pressure difference receiver as it is found in field crickets is probably the most complex one described so far, with a modified tracheal system where three important sound pressures interact at the anterior tympanum: the external sound pressure, the internal sound pressure from the ipsilateral spiracle via the leg trachea to the tympanum, and a second internal component originating from the contralateral spiracle. The tracheal connection between both sides includes a double membrane (septum) at the midline enhancing the time delay in the internal interaural transmission (Löhe and Kleindienst 1994; Michelsen and Löhe 1995). These three sound components need to interact with the proper amplitude and phase relationship to guarantee a frequency-dependent directionality at the low carrier frequencies employed by most crickets. How did such a complicated biophysical device evolve? Its frequency dependency poses another problem to the evolution of hearing, namely to match the best frequency of directionality with the frequency sensitivity of the ear: ideally both should be tuned to the CF of the male calling song. Comparative data show that a mismatch between the sensitivity and directionality tuning is not uncommon in crickets, and that independent variation of both filters is possible (Kostarakos et al. 2009).

By shifting their attention away from the commonly studied field crickets to the rich variety of other cricket species Schmidt et al. (2011) and Schmidt and Römer (2011) described cases where both the sharpness of frequency tuning and directional tuning was enhanced, its mismatch reduced, and increased IIDs were recorded. This appears to be the result of a high selection pressure of species competing for the acoustic communication channel in the nocturnal rainforest (Römer 2013). Given that the anatomical arrangement of the acoustic tracheal system provides the basis for the pressure difference receiver a comparative analysis of 40 species from three different superfamilies sheds some light on its evolution (Schmidt and Römer 2013). One of the most conspicuous features is related to modifications of the transverse trachea providing the contralateral input to the internal surface of the tympanum. The most basic form was found in a member of the superfamily Rhaphidophoridae, a primary non-hearing species *Troglophilus neglectus*, with no transverse trachea at all. Three Gryllacrididae species have an unspecialized connecting trachea with no acoustic vesicle and septum. Within the Gryllidae, an impressive anatomical transformation has taken place with the appearance of an acoustic vesicle. A striking structural modification is a double acoustic vesicle in rainforest species where large IIDs and almost perfect match between directional and sensitivity tuning had been found before (Schmidt et al. 2011).

However, demonstrating the striking variability in the anatomical structures of the acoustic tracheal system is only the first step in reconstructing the evolution of the pressure difference receiver in crickets. Relevant time and phase shifts leading to constructive and destructive interference at the tympanal membrane will depend on numerous parameters such as spiracle opening (sound entrance), cross-section and length of tracheal branches (i.e., transverse and leg trachea), their relative position to one another, the size of the acoustic vesicle and biomechanical properties of the medial septum (Fletcher 2007). Currently we know very little about the contribution of each of these parameters for the directionality and its frequency tuning, but variation of some of these factors appears to be part of the anatomical diversity seen so far (Schmidt and Römer 2013). To identify the complete apparatus for the propagation of both ipsilateral and contralateral sound components to the tympanal membrane a complete 3D-µCT analysis of these tracheal components, including quantitative measurements of tube length, volume and diameter will be necessary. These quantitative data may then be used in a modeling approach.

## Directionality in the third dimension

Although the perception of source height, i.e., elevation and depression, is certainly significant for many acoustic insects due to their habitat and lifestyle, surprisingly few studies have addressed spatial hearing in insects so far. In contrast to other taxa, where the detection of the elevation of a sound source depends either on pinnae (in mammals) or on ear asymmetries in owls (review Knudsen and Konishi 1979; Heffner and Heffner 1992), insects (and frogs) do not have ear structures specialized for the perception of elevated sound sources. Yet, as shown for the Polynesian field cricket *Teleogryllus oceanicus*, they may not be essential for spatial hearing (Wyttenbach and Hoy 1997). The authors used the ultrasound avoidance response of flying crickets as part of their startle/avoidance behavior (Moiseff et al. 1978), and determined the minimum audible angle as a measure of spatial auditory acuity. But in comparison to the acuity in azimuth, which was 11.25° from frontal and 45° from lateral, the acuity was much reduced in elevation (45° in the front and rear, and 60° to 90° from lateral).

The task (and success) in the avoidance response of a flying cricket is just to fly away from the ultrasound stimulus, but most likely not *exactly* into the opposite direction, so that such acuity is sufficient for an adaptive behavior. However, we would expect a better three-dimensional acuity when it comes to positive phonotaxis. This is evident in a behavioral study by Müller and Robert (2001) with the remarkable phonotactic accuracy of the parasitoid fly *O. ochracea* when locating its host during flight. Using sophisticated techniques to follow freely flying female flies towards the target broadcasting the hosts calling song, the authors identified three distinct phases: a brief takeoff phase; a cruising phase in which course and altitude remain quite constant; and a terminal landing phase. The terminal phase starts when the angle of source depression is about 75–90° (Müller and Robert 2001; their Fig. 3a). The flies landed quite close to the target, revealing remarkable phonotactic accuracy. Even more surprising was the finding that the acoustic stimulus could be interrupted when the fly was on its way to the loudspeaker, but the fly still initiated the final descent phase correctly and landed close to the now silent loudspeaker. This suggests that the fly had acquired enough information to navigate accurately to the sound source at the time when the stimulus was switched off. Further experiments with elevated sound sources demonstrated that the fly’s auditory system is capable of finding a sound source in the three-dimensional space, and not just on the ground. So far we do not know the proximate mechanisms enabling the small fly to perform such accurate three-dimensional localization. I therefore review findings for another acoustic insect where such information is available.

Spatial hearing is also essential for many tree crickets and katydids (and probably some grasshoppers) as well, since their microhabitats are trees and bushes, where biologically important sounds may arise from positions above and below the receiver, in addition to different locations in azimuth. Rheinlaender et al. (2007) used the duetting mode of communication between the sexes of the katydid *Leptophyes punctatissima,* (Robinson et al. 1986; Heller and von Helversen 1986; Bailey 2003) to attract phonotactically orienting males through an artificial, three-dimensional grid system towards acoustic models of the female replies. Loudspeakers broadcasting these replies were positioned either at an elevated or depressed location (by 45° each) relative to the starting point, or in the azimuth. The grid system was designed so that each point in space could be reached by the male with almost equal probability, and did not favor any phonotactic path. All males tested reached the three speaker positions in space with only little deviation from the shortest possible path, and there was no statistical difference in quantitative measures of phonotaxis towards the elevated, depressed or horizontal speaker.

A further attempt to investigate spatial hearing in the same katydid species was made using a walking compensator (Ofner et al. 2007), which allowed correlating the degree of stimulus elevation from 0 (azimuth) to 90° (exactly above the insect) with quantitative measures of the orienting movement. With increasing loudspeaker elevation the males meandered more, and there were more turns to the wrong side with increasing loudspeaker elevation. However, most males performed phonotaxis with a high acuity up to an elevation of 60°; some individuals were very accurate in their approach even at a source elevation of 75°. When males were tested in response to sound sources elevated by 90° (no binaural cues available), they circled strongly under the sound source and deviated much more from the axis of loudspeaker orientation compared to smaller elevation angles.

**Fig. 2:**
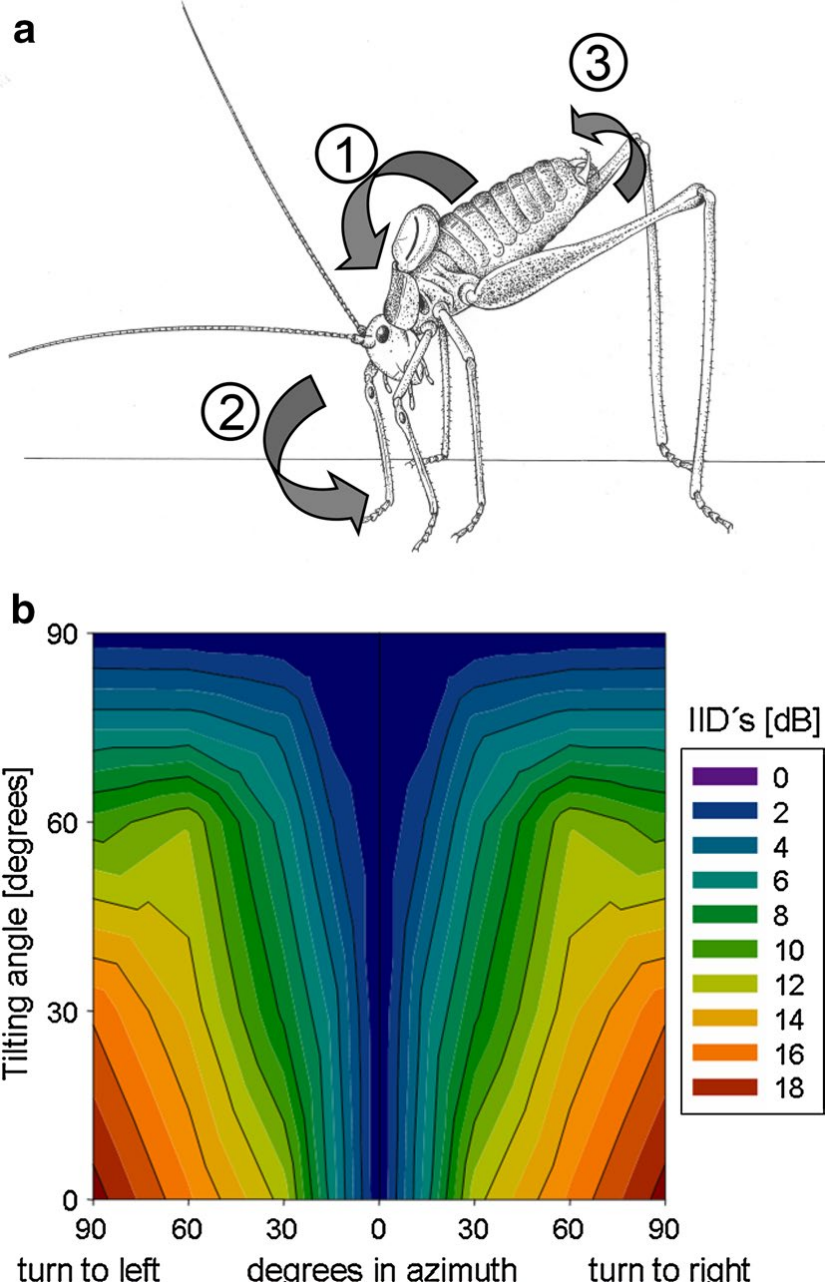
**a** Reconstruction of the body posture of males of *L. punctatissima* while they exhibit the tilting behavior. The* numbered arrows* indicate (*1*) the torque movement, (*2*) turning on the spot with a certain yaw angle and (*3*) a roll of the body axis to either side (from Ofner et al. 2007). **b** Color-coded IIDs in the peripheral auditory system of *L. punctatissima* created by a male turning to *right* or *left* at different tilting (head and thorax down) angles, with the sound source at a frontal position in the horizontal plane (from Kostarakos et al. 2007)

A peculiar behavior was observed with males under poor directional cues: males tilted their head and thorax in a forward direction, often associated with a shift of the longitudinal body axis by up to 30° to each side and also bending the dorso-ventral axis to left and right (Fig. 2a). The authors interpreted this behavior as a kind of directional scanning, similar to that of vertebrates moving their heads to localize a sound source. By doing so they might actively induce changes in binaural directional cues (see below and Fig. 2b). This is based on the fact that the main acoustic input to the ear is via the acoustic spiracle and leg trachea, guiding in particular high-frequency sound to the inner side of the ear (Michelsen et al. 1994; Michelsen 1998).

To test this hypothesis, Kostarakos et al. (2007) determined the peripheral spatial directionality of the ear using physiological methods. Whereas maximal IIDs of 18 dB occurred between ipsi- and contralateral stimulation in the azimuth, these values decreased in a more or less systematic fashion with increasing elevation or depression of the sound source, so that the gradient of IIDs could be used by the male for his directional decisions during phonotaxis. Imagine a male approaching an elevated female from some distance, the perceived elevation angle might be 30°, and by regularly meandering by 30° in the horizontal plane, this would create IIDs of about 8 dB. Approaching in the horizontal plane, the perceived elevation angle will increase with decreasing distance, and the same meandering will create smaller IIDs of 4 dB at a source elevation angle of 75°. Only if the male follows the source elevation by climbing up, he will be able to operate with the largest possible IIDs for a given turn angle.

How can the male solve the problem of ambiguity in peripheral directional cues, since depressed and elevated sound sources may result in almost the same IIDs? Two possible solutions have been suggested: one requires some kind of memory, by comparing the previous IID with the current one, so that a false decision, e.g. to climb down may result in a much more elevated angle, and corresponding smaller IIDs. Such information could be used to correct his path and climb upwards to the source the next time. The core of this model for spatial orientation is the suggestion, that it requires a sequential analysis of binaural cues, thus involving memory. This is supported by a study on the lesser wax moth *Achroia grisella* (Greenfield et al. 2002), where all interaural acoustic differences for females orienting toward the male advertisement call have been experimentally removed. Their findings also suggest that receivers may adjust their phonotactic movement in accordance with a sequential comparison of auditory input. In the case of the parasitoid fly orienting so precisely towards a sound source that has been switched off before (see above) some kind of memory must be involved as well, but of course different from the one involving sequential analysis; both deserving more attention in the future.

The second possibility for the approach of the katydid *L. punctatissima* is to avoid the wrong direction in the case of ambiguous information, by performing acoustic scanning beforehand. Figure 2 illustrates this effect for the tilting (head down) component, with a sound source fixed in the horizontal plane of the male. Largest changes in IIDs occur when the male turns right or left and does either not tilt its body or by only 30°. However, there is a substantial loss in directionality with tilting angles between 60 and 90°. The opposite would occur with a depressed sound source, because tilting would bring the source closer to the males’ horizontal plane, and thus would create large IIDs.

IIDs have to be translated into binaural discharge differences of neurons to be processed by the auditory pathway. Simultaneous recordings of the activity of a pair of directionally-sensitive interneurons allowed Kostarakos et al. (2007) determining such binaural discharge differences in response to the female reply exactly under the same conditions as experienced by males performing phonotaxis in the artificial grid system and on the walking belt (Rheinlaender et al. 2007; Ofner et al. 2007). Such discharge differences are large and reliable in the azimuth with lateral stimulation, and decrease gradually with more frontal stimulation, and also with elevation and depression of sound sources. Notably, at 60 or 75° elevation, these differences decreased to only one AP/stimulus. We should remember that at 75° elevation on the walking belt, the spatial acuity of most males strongly declined, but some males appeared to use such small bilateral differences for reliable phonotaxis. All together, these data demonstrate that the spatial acuity of the katydid is quite remarkable considering that the acoustic stimulus available for the orienting male is extremely short (1 ms) and the number of receptor cells that are involved in the processing of directional information is rather small. Moreover, receptors respond to such a short stimulus with only one action potential/stimulus, once it is above threshold (Hardt 1988). Thus, there is neither a dynamic intensity response function of a single receptor, nor is graded information available to changing stimulus angles. This is not a peculiar property of receptors in this species; the intensity response curve of auditory receptors in other insects would be similar in response to a 1-ms stimulus. Note also that the phasic response of receptors is due to the short stimulus duration, and not an intrinsic property, as in the case of the *Ormia* fly (see above). The only remaining information is the number of afferents being activated, as a result of their absolute sensitivity and/or tuning (Hardt 1988).

## Directional hearing under natural outdoor conditions

Since the pioneering work by Morton (1975) and Wiley and Richards (1978) there is an increasing awareness in the literature on acoustic communication about the properties of the transmission channel for sound signals, which may affect their amplitude, frequency spectrum and temporal pattern, in addition to masking noise which may decrease the signal-to-noise-ratio (SNR) for detection (Brumm 2013). As a result, the broadcast signal may differ substantially from the one available for a receiver at some distance. At the same time, however, the directional cues of sound under natural conditions have been largely neglected. In the previous section we have learned that some field crickets or parasitoid flies show hyperacute directional hearing when tested on a trackball under open-loop conditions in the laboratory, and that IIDs as small as 0.5–1 dB, or physiological ITDs of only 0.5–1 ms may be sufficient for reliable directional responses. Little is known about how well such cues are preserved after transmission of the signal in natural habitats. The technical difficulties in measuring such cues in the field let Rheinlaender and Römer (1986) develop a so-called “biological microphone” (adapted from Roeders pioneering work on moth hearing the echolocation calls of bats; Roeder 1967). By recording the activity of a pair of directionally-sensitive interneurons in the natural habitat of a katydid they could show that (1) directional information may be provided at remarkable communication distances, but (2) that directionality strongly depends on the spatial configuration between sender and receiver. Notably, they found positions in the habitat where the animal could detect the signal, but was unable to localize it.

A similar attempt was used to study the directionality in various types of habitat for short-horned grasshoppers (Gilbert and Elsner 2000). Using long-term recordings of tympanal nerve activity in a portable electrophysiological preparation in *Chorthippus biguttulus*, the directional dependence of the activity was monitored first in the laboratory under free field conditions, and then in various natural habitats of the species. On gravel and in sparse vegetation, the general patterns of directionality were quite similar to those in the free sound field of the laboratory, although the amount of IIDs calculated from the nerve activity was reduced. Strongest differences were found in dense vegetation, both with respect to the maximum IIDs and the location of the minima in nerve activity, which not always occurred on the contralateral side, as is typical for the free sound field.

Field crickets live and communicate on the ground, which may create specific problems for communication. Kostarakos and Römer (2010) examined directional hearing of crickets under these conditions again using a neurophysiological approach, by monitoring the activity of a pair of directionally sensitive, bilaterally homologous neurons. One major finding was again, that the directional information encoded in these neurons varied with distance, but there was no simple directional gradient on the transmission channel. Moreover, when they analyzed the consistency of the neuronal directional cues over time, they found large variations in the amount of directionality within a time window of a minute, so that the binaural discharge difference at the same receiver position varied for example from 6 to 30 APs over time. Similar strong variations occurred with respect to latency differences (Kostarakos and Römer 2010). These approaches provided direct experimental evidence that directional sensitivity strongly depends on the spatial configuration between sender and receiver, and it is not only an inherent property of the insect’s auditory system, as suggested from laboratory experiments. If directional cues under real world conditions are distorted as these data suggest, how do insects then perform in behavior? Since most data on localization behavior, and the underlying biophysics and neurophysiology are available for the field cricket *G. bimaculatus*, I will compare the localization performance of this species under outdoor conditions with the one in the laboratory.

Apparently, this performance is different in the various behavioral paradigms used for quantifying the acuity of localization. Data from compensated walking on a closed-loop trackball system, as well as orientation in a Y-maze indicated that field crickets are unable to discriminate the side of sound incidence within a frontal “area of uncertainty” covering ±25° in azimuth (Weber et al. 1981; Rheinlaender and Blätgen 1982; Weber and Thorson 1989), or 10–14° (Bailey and Thomson 1977; Oldfield 1980). However, the acuity of phonotaxis in female *G. bimaculatus* on an open-loop trackball system was much higher (Schöneich and Hedwig 2010). Their biophysical and neurophysiological measurements revealed a reliable gradient of IIDs with a slope of 0.4 dB/° between 0 and 30°, also reflected in bilateral differences of afferent nerve responses. The authors concluded that under ideal conditions these crickets achieve directional hyperacuity that rivals best directional hearing in mammals and birds, or the one reported for the fly *O. ochracea* (Mason et al. 2001).

In their natural grassland habitat female crickets reliably approach the target speaker broadcasting a standard model of the calling song in a no-choice trial, although for the shortest possible straight path females walked a considerable detour (on average about 1 m for the shortest straight path of 2 m; Hirtenlehner and Römer 2014). Such detour is significantly higher outdoors, when compared with similar experiments in a laboratory arena. Apart from the discontinuous gradients in intensity and directionality outlined above, one further reason for such differences between lab and outdoor experiments is the physical nature of the grassland in which females have to move and orient. Even if females perceive reliable sensory information about the location of a stimulus, they might be forced by dense patches of grass or larger obstacles on the ground to move into another, even wrong direction. Michelsen and Rohrseitz (1997) provided a possible explanation for the fact that field crickets perform reasonably well under such conditions. They quantified the degradation of directional cues in a grassland habitat by measuring the sound amplitude and phase close to the ears of grasshopper carcasses for different directions of sound incidence with probe microphones. The degradation increased with frequency and distance from the sound source and with distance from the ground. Amplitude cues appeared to degrade much faster with distance than phase cues. Thus, the cricket ear as a pressure difference receiver exploiting these phase cues may be particularly suited overcoming the degradation of directional cues.

The distorted directional cues under outdoor conditions are also evident when we compare results of two-choice experiments conducted in the lab and field (Hirtenlehner and Römer 2014). In general, for a significant choice larger differences in SPL, CF or call rate are necessary in the field. As expected, the open-loop trackball system requires the smallest differences, with experiments in laboratory arenas in between (for differences in SPL: 5, 1 and 3 dB, respectively). Clearly, female crickets and other acoustic insects benefit from an auditory system with high directional precision under unfavorable outdoor conditions.

## Directional hearing beyond mate finding or escaping predators: auditory scene analysis

As evident from the previous sections, the issue of directional hearing is always strongly associated with the two important tasks for a receiver, namely to use directional information for approaching towards a mate, or in the case of a predation, to initiate appropriate escape responses away from the predator. We often forget, however, that directional hearing also contributes to auditory scene analysis, by separating sound sources according to their spatial location, as well as in reducing the effect of masking, by so-called spatial release from masking.

For auditory scene analysis, a receiver groups and segregates all incoming sounds either according to their similarity or their differences in a number of sound parameters, one of which is their location in space (Bregman 1990). Signaling in acoustic insects often occurs in groups (choruses) of many individuals of the same and/or different species with the result that a complex sonic and ultrasonic background exists over which individuals must then communicate. Field measurements in populations of the katydid *T. viridissima* revealed that the problem for a receiving insect may be quite complex, requiring them to discriminate the individual calls of up to four nearby males and more than ten others within hearing range, some of which are quite similar in amplitude (Römer and Krusch 2000). How are these different acoustic objects represented in the CNS of a receiver, and how does directional hearing facilitate the separation of sound sources?

One result was that the auditory world of the katydid is sharply divided into two hemispheres, with a pronounced separation along the longitudinal body axis. Even with an angular separation of only 7.5° off the midline axis, two sound signals are better represented in the activity of the respective ipsilateral neuron. The strength of separation is given by the peripheral directionality of the ear, and by enhancement as a result of lateral inhibition. This mechanism, referred to as spatial release from masking, can also improve the detection and discrimination of signals in noise when the masker is spatially separated from the signal (Klump 1996). Again; it is based on the directionality of the receivers hearing system and contributes to the segregation of sound sources. It can improve speech perception in human listeners (Freyman et al. 2001; Bregman 1990) and the detection and discrimination of conspecific signals from heterospecifics in anurans; for a review in vertebrates see Bee and Micheyl (2008). Schmidt and Römer (2011) studied spatial release from masking for two species of rainforest crickets, which suffer from high nocturnal background noise. Laboratory experiments yielded an average signal-to-noise-ratio (SNR) of −8 dB, when masker and signal were broadcasted from the same side (owing to the sharp tuning to the CF of the conspecific signal). However, displacing the masker by 180° from the signal improved SNRs by further 6–9 dB. Surprisingly, experiments carried out directly in the nocturnal rainforest yielded SNRs of about −23 dB compared with those in the laboratory with the same masker, where SNRs reached only −14.5 and −16 dB in both species. The authors conclude that conventional speaker playbacks in the lab do not properly reconstruct the masking noise situation in a spatially realistic manner, since they use playbacks of natural or artificial noise and broadcast it to either one or the other auditory side, whereas under real world conditions the same noise is spatially separated, with multiple sound sources affecting a receiver from different directions in the azimuth and elevation/depression. Thus, without knowledge of the receiver properties and the spatial release mechanisms the detrimental effect of noise may be strongly overestimated. The contribution of the peripheral directionality of the ear and additional central nervous bilateral interactions (through lateral inhibition) for spatial release from masking is currently unknown.

## Abbreviations

AP: Action potential
SPL: Sound pressure level
IID: Interaural intensity difference
ITD: Interaural time difference
SNR: Signal-to-noise-ratio
